# Cardiac amyloidosis: evolving pathogenesis, multimodal diagnostics, and principles of treatment

**DOI:** 10.17179/excli2023-6284

**Published:** 2023-08-03

**Authors:** Gnana Deepthi Medarametla, Ripudaman Singh Kahlon, Lampimukhi Mahitha, Sanobar Shariff, Naga Praneeth Vakkalagadda, Hitesh Chopra, Mohammad Amjad Kamal, Neil Patel, Yashendra Sethi, Nirja Kaka

**Affiliations:** 1 Pranavi Children’s and Eye Hospital, Kandukuru, Andhra Pradesh, India; 2 Pear Research, Dehradun, India; 3 Government Medical College, Amritsar, Punjab; 4 Rangaraya Medical College, Kakinada, Andhra Pradesh, India; 5 Yerevan State Medical University, Yerevan, Armenia; 6 Guntur Medical College, Guntur, Andhra Pradesh, India; 7 Department of Biosciences, Saveetha School of Engineering, Saveetha Institute of Medical and Technical Sciences, Chennai, Tamil Nadu-602105, India; 8 Institutes for Systems Genetics, Frontiers Science Center for Disease-Related Molecular Network, West China Hospital, Sichuan University, China; 9 King Fahd Medical Research Center, King Abdulaziz University, Saudi Arabia; 10 Department of Pharmacy, Faculty of Allied Health Sciences, Daffodil International University, Bangladesh; 11 Enzymoics, 7 Peterlee Place, Hebersham, NSW 2770; Novel Global Community Educational Foundation, Australia; 12 GMERS Medical College, Himmatnagar, Gujarat, India; 13 Government Doon Medical College, Dehradun, Uttarakhand, India

**Keywords:** cardiac amyloidosis, ATTR amyloidosis, gene therapy, amyloid, cardiomyopathy, CRISPR

## Abstract

Amyloidosis is a protein deposition disorder in which insoluble fibril structures accumulate in the bodily tissues damaging the organ function. Cardiac amyloidosis is a severe but under-reported medical condition characterized by the accumulation of amyloid in the extracellular area of the myocardium, which results in thickening and stiffening of ventricular walls. Cardiac amyloidosis has recently gained much attention with its slowly surging incidence. With this study, we seek to comprehensively compile the pathophysiology and clinical picture of cardiac amyloidosis subtypes, extending a clinically oriented, up-to-date clinical approach to diagnosis and therapy. Cardiac amyloidosis can be caused by rare genetic mutations which may be inherited or acquired. The growing incidence can be attributed to advancements in imaging methods and other diagnostic modalities. Most occurrences of cardiac amyloidosis result from two forms of precursor protein: transthyretin [TTR] amyloid and immunoglobulin-derived light-chain amyloid. Prompt identification of cardiac amyloidosis can facilitate the implementation of evolving therapeutic interventions to enhance the outcomes. The modalities for the management of CA have evolved significantly in the last ten years. Apart from therapies for modifying disease and heart failure, a myriad of novel therapeutic approaches that target specific aspects of the disease, including gene therapies, are being researched. These aim at impeding its progression and improving clinical outcomes.

See also Figure 1(Fig. 1).

## Introduction

The abnormal accumulation of insoluble polymeric fibrillar proteins outside the cells (in tissues, blood vessels, and organs) is the hallmark of amyloidosis (Wechalekar et al., 2016[136]). Amyloidosis, as a systemic disease, often involves several body organs - showing varied clinical presentations. Cardiac amyloidosis can be attributed to mutations found in TTR protein resulting in decreased stability and transthyretin (ATTR) misfolding. It can also be caused due to clumping of immunoglobulin light-chain (AL) (Wechalekar et al., 2016[136]).

Transthyretin, otherwise known as prealbumin, has a physiological role in facilitating the transport of both retinol-binding protein and thyroxine. It is predominantly produced in the liver as a homotetrameric protein circulating in the body (Saelices et al., 2015[118]). Transthyretin is further categorized into wild-type (ATTRwt) and hereditary type (ATTRv). There is a growing prevalence of ATTR disease and is often identified as a leading cause of heart failure among elderly patients who exhibit restrictive physiology or left ventricular hypertrophy due to the deposition of amyloid (Maurer et al., 2017[86]; Emdin et al., 2019[36]; Manolis et al., 2019[81]; Oerlemans et al., 2019[99]; Ruberg et al., 2019[115]; Yamamoto and Yokochi 2019[142]; Griffin et al., 2021[51]). ATTR-CM affects up to 16 % of individuals who need valve replacement due to aortic stenosis with an average age of 83.7 years. Additionally, it has been seen to affect approximately 13 % of people over the age of 82, who suffer from heart failure with preserved left ventricular ejection fraction. And around 8.2 % of 60-year-olds who have hypertrophic cardiomyopathy tend to be diagnosed with ATTR-CM (Gladden et al., 2014[47]; González-López et al., 2015[49]; Treibel et al., 2016[133]; Castaño et al., 2017[18]; Ternacle et al., 2019[131]; Maurizi et al., 2020[89]). In post-mortem studies, it has been found that a considerable proportion of older individuals may exhibit amyloid build-up in cardiac tissue, which originates from plasma transthyretin. The prevalence of this occurrence can range up to 25 %^. ^(Cornwell et al., 1983[21]; Tanskanen et al., 2008[130]). Free radical accumulation and cell apoptosis are also associated with TTR amyloid deposition (Zhang et al., 2013[143]; Wieczorek and Ożyhar, 2021[140])

ATTR-CM typically has an insidious onset with mild symptoms, but unchecked progression can have a poor prognosis (Ruberg et al., 2019[115]). At the point of diagnosis, it is possible to determine the National Amyloidosis Center ATTR stage by utilizing both N-terminal pro-B-type natriuretic peptide concentration and estimated glomerular filtration rate (eGFR). The prognosis of ATTR patients can be determined based on this staging (Gillmore et al., 2018[45]; Cappelli et al., 2020[14]) . The rise of ATTR amyloidosis diagnosis has recently been attributed to cardiac magnetic resonance imaging and repurposed bone scintigraphy (Fontana et al., 2014[40]; Maurer, 2015[84]).

Several new therapies are being developed at the moment, including patisiran, a gene-silencing RNA therapy, and Inotersenan antisense oligonucleotide, which are inhibitors of TTR protein production, and tafamidis, a drug that helps preserve the soluble non-amyloid conformation of the TTR protein. (Adams et al., 2018[1]; Benson et al., 2018[6]; Maurer et al., 2018[88]; Kristen et al., 2019[68]). Contemporary treatment strategies that reduce the expression of TTR or maintain TTR stability and the RNA interference therapies inhibiting TTR synthesis (Adams et al., 2018[1]; Benson et al., 2018[6]) (Bulawa et al., 2012[11]; Berk et al., 2013[8]; Macedo et al., 2020[79]) are effective in impeding or completely stopping the advancement of this condition. Stabilization of TTR improves survival in ATTR-CM patients (Maurer et al., 2018[88]). This review provides an updated summary of the natural history of ATTR-CM, pathogenesis, clinical presentations and variants, imaging techniques, diagnostic modalities, and recent advances in treatment modalities.

## Clinical Presentation

The classification of cardiac amyloidosis is determined by the amyloid protein type, which divides it into three variants: AL (immunoglobulin light chain) amyloidosis, ATTRwt (previously known as "senile cardiac amyloidosis"), and ATTRM/ mutated ATTR that can result in hereditary cardiac amyloidosis.

The frequency of cardiac manifestation is different in different variants of amyloidosis. The AL variant, also called primary amyloidosis, is the most frequent among these, in which approximately 90 % of the patients show cardiac presentation. The rarest to show cardiac presentations is the SA variant or secondary amyloidosis. In ATTRwt, the heart typically presents as the only organ demonstrating clinical manifestations.

In hereditary ATTR, both cardiac and neurological presentations dominate (Maurer et al., 2019[85]). Individuals diagnosed with ATTRwt may exhibit a higher incidence of irregular heart rhythms compared to those who have hATTR (Maurer et al., 2016[87]). All types of amyloidosis eventually cause restrictive cardiomyopathy with preserved diastolic dysfunction presenting as heart failure with preserved ejection fraction (HFpEF) (Maurer et al., 2019[85]). Symptoms of cardiac amyloidosis are predominantly associated with right heart failure. The clinical manifestations of AL amyloidosis are typically characterized by rapid progression, whereas ATTRw or senile amyloidosis presents with slowly progressing signs and symptoms and mainly presents in old age. The clinical picture is mostly of heart failure-exertional dyspnea and pain in the chest typical or atypical for angina pectoris may or may not be present. In the advanced stages of the illness, orthopnea is a common symptom. Paroxysmal nocturnal dyspnea, fatigue, presyncope symptoms, or syncope can also occur. Other right heart failure symptoms like peripheral edema, ascites, hepatosplenomegaly, and pleural effusion can also occur (Gertz et al., 2015[42]; Bhogal et al., 2018[9]). Restrictive cardiomyopathy can also lead to arrhythmias and conduction abnormalities. On examination, distant heart sounds, raised jugular venous pressures, and pulses paradoxus can be observed. Third heart sound may be heard in advanced disease. Kussmaul's sign may be present but is not pathognomonic of cardiac amyloidosis. The blood pressure tends to be generally low and can decrease even further when standing up, especially in the presence of autonomic neuropathy (Kilpatrick et al., 1967[64]).

In most patients, cardiac manifestations occur late and are preceded by extracardiac manifestations. The extracardiac manifestations can aid in the early diagnosis of this vastly underdiagnosed condition. In AL amyloidosis most common findings are macroglossia (almost pathognomonic but present only in 10 % of cases) (Guidelines Working Group of UK Myeloma Forum, 2004[52]), periorbital purpura (panda eyes), and nail dystrophy (Rubinow and Cohen, 1978[116]). In senile and hereditary amyloidosis, most have a neuropathy dominant picture-carpal tunnel syndrome, tendon rupture, and ascending symmetrical length-dependent sensorimotor axonal polyneuropathy (Dubrey et al., 2011[32]). Sudden death in amyloidosis occurs primarily due to cardiac causes, out of which the most common is electromechanical dissociation (Cueto-Garcia et al., 1984[24]). Ventricular arrhythmias are rare, but atrial rhythm disturbances are prevalent, consequently enhancing the likelihood of atrial fibrillation leading to the thromboembolic phenomenon.

## Pathophysiology

Amyloid formation occurs when a protein loses its normal functional fold or fails to adopt it correctly. Various factors contribute to protein misfolding and aggregation, including abnormal proteolysis, point mutations, and post-translational modifications such as phosphorylation, oxidation, and glycation. The misfolded protein or peptide subsequently interacts with other misfolded proteins or peptides, leading to the formation of oligomers. These oligomers circulate in the bloodstream and eventually deposit as well-structured fibrils in the interstitial spaces of specific target organs (Westermark et al., 1990[138]). 

### Systemic amyloidosis

Systemic amyloidosis is categorized into three main types: (a) Primary systemic amyloidosis, also known as plasma-cell dyscrasia, (b) Amyloidosis associated with multiple myeloma, and (c) Secondary systemic amyloidosis resulting from chronic inflammatory or infectious conditions. In primary systemic amyloidosis, insoluble deposits of monoclonal immunoglobulin (Ig) light (L) chains or L-chain segments accumulate in various tissues and structures, including smooth and striated muscles, connective tissues, blood vessel walls, and peripheral nerves (Zhao et al., 2013[146]).

### Cardiac amyloidosis

**Figure 2:**
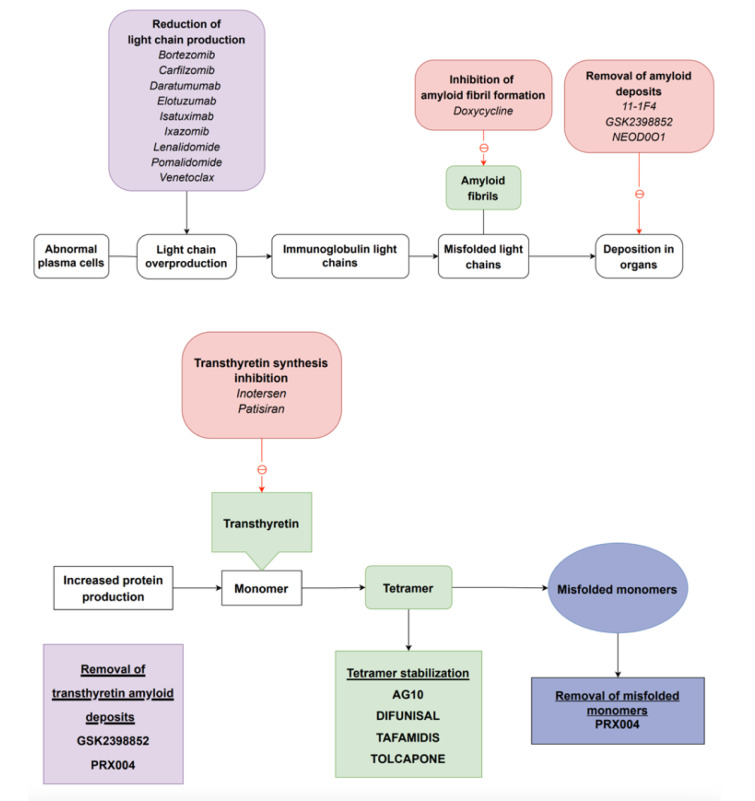
Pathophysiology and common drug targets

Cardiac amyloidosis is a condition characterized by the deposition of amyloid in the parenchymal tissue of the heart, leading to a restrictive pathology and resulting in diastolic dysfunction. This extracellular amyloid deposition disrupts the tissue structure mechanically and induces proteotoxicity, inflammation, generation of reactive oxygen species, apoptosis, and autophagy (Buxbaum et al., 2012[13]). The infiltration of amyloid proteins contributes to the development of atrial fibrillation, and even in the presence of sinus rhythm, there is an increased risk of atrial thrombus formation and thromboembolism. In AL disease, amyloids can accumulate within or around the small arterioles of the heart, which may lead to symptoms such as angina or myocardial infarction. The pathophysiology has been summarized in Figure 2(Fig. 2).

### ATTR (Transthyretin amyloidosis)

The liver produces a plasma transfer protein called transthyretin (Westermark et al., 1990[138]). It functions in the transfer of thyroid hormone and retinol and circulates as a stable tetramer. It becomes unstable as it ages or mutates, and it separates into oligomers and monomers, which causes organ failure either by building up as amyloid fibrils or being directly toxic. The two main categories of ATTR are Wild type and Hereditary type. The heart, ligament, thyroid, kidneys, peripheral nerves, and lungs are the main organs the wild variety damages. Although the exact cause of tetramers' dissociation with age is unknown, it is hypothesized that it may be brought on by post-translational biochemical alterations in TTR (Zhao et al., 2013[146]) or chaperone proteins in the liver (Buxbaum et al., 2012[13]). Aging causes a general rise in protein oxidation. TTR that had undergone oxidation was found to be less robust than unoxidized TTR and to be more prone to aggregate and fibril formation (Zhao et al., 2013[146]). Recently, it has been proposed that ATTR fragmentation brought on by proteolysis is a process that promotes the growth of ATTR amyloidosis (Si et al., 2021[124]). ATTRv (inherited ATTR) is an autosomal dominant hereditary systemic amyloidosis (Benson et al., 2020[5]). More than 150 TTR gene variants have been discovered, most of which are amino acid mutations caused by a single nucleotide substitution (Rowczenio et al., 2014[113]). 

ATTRv amyloidosis presents with various clinical phenotypes, including primary polyneuropathy associated with the V30M mutation, cardiomyopathy linked to the V20I, V122I, L111M, and I68L mutations, and a mixed phenotype observed in cases of the E89Q and T60A mutations (Rapezzi et al., 2011[108]). Among these, the V122I mutation in the TTR gene is the most commonly found amyloidogenic mutation worldwide and is predominantly associated with familial cardiomyopathy in individuals of African descent (Jacobson et al., 1997[58]). On the other hand, the S52P variant is associated with aggressive and highly penetrant systemic amyloidosis (Mangione et al., 2014[80]).

AA amyloidosis, dialysis-associated amyloidosis (the precursor protein is beta 2 microglobulin), and isolated atrial amyloidosis (due to fibrils deposits from atrial natriuretic factor) are other less prevalent forms of CA. Along with inflammatory bowel disease, rheumatoid arthritis, and hidradenitis suppurativa, AA amyloidosis is linked to other auto-inflammatory diseases (Welzel et al., 2021[137]).

### Evolving molecular mechanisms

**Protein Unfolding and Misfolding:** Proteins have a perfectly folded, three-dimensional conformation that keeps their quantity and function intact. Protein folding happens spontaneously in accordance with the amino acid sequence following the endoplasmic reticulum's synthesis of the protein polypeptide chain (Dobson, 2003[26]). Chaperones are necessary for proteins to retain the proper folding structure. Oxidation, low pH, and high temperature are just a few examples of extracellular triggers that cause the 3-dimensional structure to be disrupted and polypeptide chains to unfold. 

Typically, misfolded proteins undergo degradation and elimination by the proteasome (Wickner et al., 1999[139]). However, certain misfolded proteins are released extracellularly and undergo reassembly into a 3-dimensional conformation that is characterized by a high content of β-sheets (Merlini and Bellotti, 2003[90]). These misfolded proteins then polymerize with other misfolded proteins, resulting in the formation of amyloid fibrils. During this process, temporary protein unfolding exposes previously concealed hydrophobic residues, which are subsequently targeted and cleaved by proteases. This proteolytic cleavage further destabilizes the proteins, accelerating the misfolding and aggregation process. In the case of variant transthyretin amyloidosis (ATTRv), a change in the amino acid sequence (caused by a single-point mutation in the genome) can make partial unfolding or misfolding more likely to occur. Additionally, factors such as defects in protein biosynthesis regulation, oxidation and deamidation of amino acid side chains, chemical modifications of polypeptide chains, binding of metal ions, and other mechanisms have been observed to increase the propensity for protein misfolding (Kapurniotu 2011[62]).

### Nucleation-dependent polymerization

The process of amyloid fibril formation can be divided into two stages: nucleation and subsequent elongation.

**Nucleation phase**: During the nucleation phase, the initial nucleus of amyloid fibrils is formed as misfolded protein monomers assemble into a soluble oligomer. This process of nucleation is not spontaneous but rather requires specific conditions. The nucleation phase plays a crucial role in regulating the overall rate of fibril formation. 

**Elongation phase**: Once nuclei have formed, the subsequent elongation phase occurs, characterized by the sequential binding and growth of monomers, also known as "nucleation-dependent polymerization." This allows amyloid fibrils to propagate and extend their structure along the fiber axis (Naiki and Gejyo, 1999[96]). Common structural features of amyloid fibrils include their non-branched morphology, typical diameter ranging from 7.5 to 10 nm, and a cross-β-sheet secondary structure (Eisenberg and Jucker, 2012[34]).

### Deposition of amyloid fibrils

Minor variations in the amino acid sequences of precursor proteins can significantly impact the organs targeted by amyloid accumulation. In the case of transthyretin (TTR) mutations in ATTRv, the specific location of the mutation influences the clinical characteristics and timing of amyloid deposition. Furthermore, the properties of target organs can change due to the fragmentation of amyloid transthyretin (ATTR) caused by proteolysis (Bergström et al., 2005[7]). The concentration of precursor proteins plays a role in determining the rate of fibril development in systemic amyloidosis. In addition to fibril seeds, the tissue environment needs to meet other requirements, such as appropriate local concentration and low pH. Factors such as metals, proteases, shearing force, and components of the extracellular matrix (e.g., glycosaminoglycans, collagen) are believed to play a crucial role in promoting aggregation and the formation of oligomers (Stevens and Kisilevsky, 2000[128]).

## Clinical Variants

In recent times, diagnoses of cardiac amyloidosis variants-ATTR amyloidosis and systemic monoclonal immunoglobulin light chain amyloidosis (AL) amyloidosis, the most frequently diagnosed form of cardiac amyloidosis-have risen. The latter is caused by the accumulation of either variant or wild-type TTR amyloid in the heart's interstitium.

**AL amyloidosis: **Accumulation of light immunoglobulin chains due to a plasma-cell dyscrasia causes AL amyloidosis. With about 2,500 to 5,000 new instances reported in the USA each year on average, it is a rare disorder. According to a recent study, the prevalence and incidence of AL amyloidosis among people aged 18 to 64 years is increasing significantly (Papingiotis et al., 2021[100]).

**ATTR amyloidosis:** A mutated gene that makes transthyretin's tetramer more prone to instability is the underlying cause of hereditary ATTR amyloidosis. A liver-produced protein called transthyretin transfers retinol and thyroid hormone. More than 120 transthyretin mutations related to amyloidosis have been found documented, with some genetic variations confined to geographical areas or ethnic groupings. Diverse transthyretin mutations have varying onset ages and cardiomyopathy risk. The Val30Met variant is the transthyretin mutation that has been described the most frequently, but the Leu111Met and Ile68Leu variants are primarily associated with a cardiac phenotype (Patel and Hawkins, 2015[102]). The Val122Ile (pV142I), which affects 3.4 % of African Americans, is the most prevalent. The next mutation is Thr60Ala (pT80A), which was first discovered in Northern Ireland and patients with this mutation have a significant probability of developing carpal tunnel syndrome (> 70 %). 

ATTR amyloidosis mainly affects older males with a cardiac predominance phenotype (Ruberg and Berk, 2012[114]). Virtually all individuals with wild-type ATTR amyloidosis are over sixty years old, and the incidence only rises with the age. Most individuals with ATTR (wild-type) are men; however, recent investigations indicate that women form a sizeable proportion of those affected. Heart amyloidosis is the primary characteristic of wild-type ATTR (Yamamoto and Yokochi, 2019[142]).

## Diagnosis

Cardiac amyloidosis is a difficult-to-diagnose disorder with a dismal outcome, but new treatments and the promise for newer drug targets are improving its prognosis. Echocardiography is generally the preferred imaging technique. If any abnormalities are detected, non-invasive techniques like cardiac magnetic resonance imaging [CMR] and nuclear imaging [NI] can be used to diagnose and evaluate it. 

For AL-type cardiac amyloidosis, it may be possible to diagnose the condition without resorting to regular cardiac imaging techniques by noting levels of NT-pro BNP or troponins which are abnormally high, provided that other potential causes of abnormally high NT-pro BNP or troponins have been eliminated. Interestingly, if the serologic markers for AL are negative and the results of the nuclear imaging tests meet the criteria, then a formal medical diagnosis of ATTR CA can be established (Wang et al., 2020[134]).

**ECG**: QRS complexes of low voltage-5 millimeters in the limb leads or 10 millimeters in the precordial leads-are usually seen as an electrocardiographic indication of CA (Ruberg et al., 2019[115]). This is a result of the loss of heart tissue caused by diffuse amyloid deposition. However, only 50 % of patients with AL-cardiac amyloidosis and 25-40 % of patients with ATTR-CA fulfil the criteria for low voltage QRS complexes (Dungu et al., 2012[33]; Mussinelli et al., 2013[94]). The thickening of the cardiac walls in amyloidosis results from the accumulation of amyloid deposits and not from true cardiomyocyte hypertrophy. Thus, QRS voltage on ECG and LV mass does not have a linear association. This implies that clinicians should not disregard the possibility of a diagnosis of CA simply due to absence of low voltage QRS voltages on an ECG (Porcari et al., 2023[106]). Around half of the individuals with AL cardiac amyloidosis may experience pseudo-infarct patterns, and their ECGs often show deficient R wave progression (Murtagh et al., 2005[93]; Ruberg et al., 2019[115]). Atrial arrhythmias, in specific atrial fibrillation, often occur in ATTR cardiac amyloidosis, likely due to the heavy infiltration of amyloid in the atria. Around 22 % of individuals with cardiac amyloidosis may also encounter an atrioventricular block. Bundle branch blocks and intraventricular conduction delays are other ECG signs in cardiac amyloidosis, which are particularly noticeable in ATTR (Huang et al., 2015[56]).

In AL amyloidosis, ECG can reveal left ventricular hypertrophy in about 10-15 % of patients, but this is probably caused by pre-existing hypertension in conjunction with amyloidosis. Conduction system diseases and Bradyarrhythmia is common, likely caused by progressive infiltration of amyloids and autonomic system dysfunction, particularly in AL and ATTRv-cardiac amyloidosis (Murtagh et al., 2005[93]; Porcari et al., 2023[106]). The ECG is a cheap, readily available test that can provide essential clues to ascertain the diagnosis, guide the further investigation, and aid decision-making.

**Multimodal imaging**: A single imaging technique cannot offer a comprehensive diagnosis or an understanding of the morphological and functional repercussions of CA. Therefore, for CA to be clinically evaluated, it is essential to use multiple imaging modes. Radioisotope bone scans have recently been used effectively to identify ATTR-CA in the absence of monoclonal proteins. Nevertheless, scintigraphy alone is insufficient to assess both the structure and activity of the CA. Therefore, even today, echocardiography is a major imaging technique for recognizing and managing CA, as it can be employed to arouse suspicion, differentiate from other illnesses, and assess the results of treatment. Magnetic resonance imaging of the cardiovascular system (CMR) can offer identification of tissue properties in CA. Consequently, these multiple imaging methods should be used together to maximize the diagnosis of CA (Huang et al., 2015[56]; Dorbala et al., 2019[28]; Jung et al., 2019[61]; Hanna et al., 2020[54]; Martinez-Naharro et al., 2020[83]).

**Echocardiography**: Echocardiography is still the leading imaging tool due to its accessibility, safety, affordability, portability, and extensive history of usage when assessing diastolic performance and cardiac deformation, though it often necessitates verification by additional tests (Nagueh et al., 2009[95]; Lang et al., 2015[71]). Imaging the heart is also essential in determining the level of risk for those suffering from cardiac amyloidosis (Wang et al., 2020[134]). Echocardiographic variables used to assess cardiac amyloidosis include ejection fraction, left ventricular size and wall thickness, echogenicity of the myocardium, diastolic function, left ventricular longitudinal strain, size and function of the atrium, assessment of interatrial septum, right chambers systolic pressures assessment, valve thickness, and pericardial effusion (Dorbala et al., 2020[29]). The detection of LVH with echocardiography is considered the primary indicator of CA. It is usually necessary before a further investigation into the condition is carried out in patients not already at risk, which often manifests as symmetric and concentric enlargement. However, asymmetric hypertrophy may additionally be detected (González-López et al., 2017[48]; Yamamoto et al., 2018[141]). 

When the left ventricle of the heart has thickened but the heart remains of normal size, in absence of other apparent causes like hypertension or hypertrophic cardiomyopathy, then cardiac amyloidosis must be considered (Gertz et al., 2005[43]). In this circumstance, an ECG with low voltage tends to suggest CA. 

There is usually a spectrum of impairment in diastolic function, varying from slight to severe, with signs of augmented left ventricular filling pressures (Lang et al., 2015[71]). It is common to observe a decrease in left ventricular global longitudinal strain despite ejection fraction remaining normal, and the so-called "cherry-on-top" pattern, which is characterized by lower strain in basal segments than the apex. This was quantitatively illustrated as minimum required ratio between the relative apical longitudinal strain and the average of mid longitudinal and basal strain to be 1, indicating cardiac amyloidosis (Phelan et al., 2012[105]). Individuals being examined with echocardiography who are ultimately identified to have CA are most likely either displaying symptoms with recognizable signs that lead to the diagnosis of CA during their assessment for congestive heart failure or are not exhibiting symptoms but are in danger of having CA due to their history of non-cardiac AL amyloid involvement or having a family with a known positive genotype for hATTR (Scheel et al., 2022[120]).

**MRI**: Magnetic Resonance Imaging has a major advantage over other forms of imaging since it can provide a detailed analysis of tissue and accurately measure the degree of infiltration. However, the need for access to the equipment, expense, fear of enclosed spaces, use of contrast dyes, and presence of metal objects may prevent its use (Wang et al., 2020[134]; Kramer et al., 2013[67]). Patients with inadequate acoustic windows on echocardiograms will certainly benefit from using cardiac magnetic resonance imaging (CMR). Furthermore, CMR's improved tagging and strain techniques enable a more comprehensive assessment of myocardial deformation than with echocardiography (Scheel et al., 2022[120]).

An investigation of 36 people with heart failure (HF) and either a myocardial biopsy or autopsy showing cardiac amyloidosis (CA) (n = 11) or additional non-cardiac biopsy along with left ventricular hypertrophy (LVH) on echocardiogram revealed that close to a third of the patients had normal left ventricular mass index (LVMI) based on cardiac magnetic resonance (CMR) (Wassmuth et al., 2011[135]). This implies that CA may be present in some cases without clear hypertrophy as assessed by LVMI on CMR. There may also be disparities in the level of LVH on CMR dependent on the type of CA, with wild-type transthyretin-related (wtATTR) generally having higher LVMI, potentially linked to the elderly demographic and prolonged asymptomatic state in comparison to amyloid light-chain-related cardiac amyloidosis (AL-CA) (Kristen et al., 2015[69]).

**CMR**: Cardiac Magnetic Resonance imaging is an effective tool for distinguishing CA from other ailments causing Left Ventricular Hypertrophy (LVH). In comparison with healthy individuals with Hypertensive Heart Disease (HHD), those with CA have been found to have a reduced Left Ventricular Ejection Fraction (LVEF) and Right Ventricular Ejection Fraction (RVEF), increased Left Ventricular Mass Index (LVMI), heightened Right Ventricular Mass (RVM), and a higher magnitude of Right Ventricular Hypertrophy (RVH) on CMR scanning (Fattori et al., 1998[37]). 

**Nuclear Imaging**: Currently, nuclear imaging is the sole non-invasive method capable of distinguishing between ATTR and AL cardiac amyloidosis (Porcari et al., 2023[106]). Nuclear imaging involves cardiac scintigraphy, which utilizes radiotracers derived from bone scintigraphy, and PET (Positron Emission Tomography) utilizing targeted tracers specific to amyloid proteins. These combined techniques form the basis of nuclear imaging for this purpose. Nevertheless, cardiac scintigraphy by SPECT is the go-to procedure for diagnosing patients with ATTR (Li et al., 2021[76]). The primary concept involves labeling a tracer with a radioactive material that has an affinity to a particular organ or health (Wassmuth et al., 2011[135]).

**Cardiac scintigraphy**: Examining pictures, both flat and SPECT is the initial step in understanding each study. If there is wide uptake of the tracer in the heart muscle, this is an indication of ATTR cardiac amyloidosis (Fattori et al., 1998[37]). In current practice the use of 99mTc-phosphate derivatives is accepted in differentiating between AL and ATTR amyloidosis. This, 99mTc-PYP (technetium pyrophosphate), includes 99mTc-DPD (technetium-3,3-diphosphono-1,2-propanodicarboxylic acid) and 99 mTc-HMDP (technetium hydroxy methylene-diphosphonate). The way that these derivatives work relates to the binding of calcium-rich TTR fibrils to tracers that are based on phosphate (Chen and Dilsizian, 2012[19]). These radiotracers bind more to ATTR-type amyloid deposits that are rich in calcium than AL-type, so they have a high level of specificity and sensitivity for ATTR amyloidosis (Wang et al., 2020[134]). The use of SPECT imaging is necessary to know if the uptake is happening in the myocardium or in other areas, such as the blood pool or other areas around the heart (Hanna et al., 2020[54]).

Genotyping and immunohistochemistry were used as the reference standard, and the positivity of the scan was evaluated by analysing the uptake using a rating system still utilized in medical practice. This rating system consists of four grades: grade 0 represents no uptake in the heart and normal uptake in the bones, grade 1 indicates lower uptake in the heart compared to the bones, grade 2 signifies similar uptake in the heart and bones, and grade 3 indicates higher uptake in the heart compared to the bones, or even the absence of bone uptake (Treglia et al., 2018[132]).

**PET**: A comprehensive research study with 1217 participants believed to have cardiac amyloidosis showed that using phosphate-based tracers through cardiac scintigraphy had positive predictive value and 100 % specificity for ATTR cardiac amyloidosis. A systematic review validated that the scintigraphy accuracy in identifying ATTR CA was over 90 % in both sensitivity and specificity (Treglia et al., 2018[132]). 18F-florbetapir, N-[methyl-(11)C]2-(4′-methylamino-phenyl)-6-hydroxybenzothiazole (11C-Pittsburgh compound B 11C-PiB), and 18F-florbetaben were the commonly used and studied tracers (Patel et al., 2021[103]). A pilot study that included 19 participants (5 without CA and 14 with CA) presented that retention of 18F-florbetapir was higher in CA patients compared to the control group. Lee et al. and Jung et al. further reported that 11C-PiB PET was helpful in diagnosing CA (Lee et al., 2015[74]; Jung et al., 2022[60]). Qualitative evaluation of the images is done by analyzing the target-to-background ratio, myocardial standardized uptake value (SUV), and myocardial retention index (Dorbala et al., 2014[30]). Nuclear imaging evaluates the degree of a disease and can measure the amyloid accumulation using the Standardized Uptake Value (SUV) semi-quantitatively (Kinahan and Fletcher 2010[65]). The Retention index (RI) is another method used to measure the amount of amyloid in the heart through PET imaging (Antoni et al., 2013[4]; Cuddy et al., 2020[23]; Kero et al., 2020[63]). This technique allows for an estimation of amyloid accumulation quantitatively. In addition, PET images can be combined with MRI or CT for better accuracy of the readings. Studies have shown that if the myocardium-to-blood pool ratio (i.e. target-to-background ratio is more than 1.5) and the retention index is above 0.025/min then it can be used to differentiate between patients with cardiac amyloidosis and healthy individuals (Law et al., 2016[72]; Manwani et al., 2018[82]) 

**Biomarkers**: Diagnosis of CA poses a significant challenge, as the clinical presentation is often non-specific, and with late onset of symptoms in the disease course. Imaging techniques have proven essential in diagnosing CA, but given the disease's progressive nature, early detection is crucial to optimizing outcomes. Biomarkers have emerged as a promising tool to aid in the prognosis and diagnosis of CA, with several studies shedding light on their role in the disease.

### Biomarkers in diagnosing AL-cardiac amyloidosis

Biomarkers such as B-type natriuretic peptide (BNP), N-terminal fragment of the pro-brain natriuretic peptide (NT-proBNP), and high sensitivity cardiac troponin have demonstrated utility in detecting cardiac involvement, predicting prognosis, and evaluating treatment response in patients with systemic immunoglobulin light chain amyloidosis (AL) (Law et al., 2016[72]; Manwani et al., 2018[82]; Kero et al., 2020[63]; Ren et al., 2021[110]). The serum-free light chain assay is preferred over serum or urine protein electrophoresis due to its enhanced sensitivity in detecting AL amyloidosis. Combining troponin blood levels with two echo-derived strain parameters has yielded a diagnostic score with a sensitivity of 94 % and specificity of 97 % for diagnosing cardiac involvement in AL amyloidosis patients (Nicol et al., 2020[97]) NT-proBNP serves as an independent predictor of death and the need for dialysis, with levels exceeding 8500 ng/L associated with hazard ratios of 3.3 for death and 3.0 for dialysis. This underscores the significance of NT-proBNP as a valuable biomarker for risk stratification in cardiorenal AL amyloidosis (Rezk et al., 2019[111]).

Hepatocyte growth factor (HGF) is significantly elevated in patients with amyloid light-chain (AL) cardiac amyloidosis compared to other groups, including those with non-cardiac systemic amyloidosis (Swiger et al., 2016[129]), and has been identified as a discriminatory serum biomarker that may improve prognostic and staging systems in AL and ATTR cardiac amyloidosis (Wang et al., 2020[134]). Interleukin-6 (IL-6) and vascular endothelial growth factor (VEGF) were not found to be significantly different in AL cardiac amyloidosis compared to other groups (Swiger et al., 2016[129]). One study found that cardiac biomarkers, namely N-terminal pro-b-type natriuretic peptide (NT-proBNP) and high-sensitivity cardiac troponin (hs-cTn), were prognostic in systemic light chain amyloidosis (AL) patients without cardiac involvement by standard criteria. The study followed 378 patients with Mayo stage I AL for a median of 42 months, during which 28 % of patients showed cardiac involvement by cardiac magnetic resonance imaging (CMRI). Age, autonomic nervous system involvement, elevated NT-proBNP (>152 ng/L), elevated hs-cTn (>10 ng/L), and CMRI-detected cardiac involvement were all associated with reduced survival, with NT-proBNP emerging as an independent predictor of mortality (Sharpley et al., 2020[123]). While these biomarkers may have diagnostic utility in distinguishing AL cardiac amyloidosis from other conditions with similar clinical or morphologic characteristics, further studies are necessary to determine their predictive value for survival (Swiger et al., 2016[129]).

### Biomarkers in diagnosing ATTR-cardiac amyloidosis

Biomarkers have a prominent role in predicting prognosis in (ATTR-CA) patients. Two biomarker-based staging systems are available, one including estimated glomerular filtration rate (eGFR) and N-terminal pro-B-type natriuretic peptide (NT-proBNP), and the other including troponin I (TnI) and NT-proBNP. A comparison of the two systems found that the NT-proBNP and eGFR systems had better prognostic accuracy and resulted in effective survival stratification, while the combination of TnI and NT-proBNP was not effective in successfully differentiating survival (Cappelli et al., 2020[14]). However, biomarkers alone are not sufficient for diagnosing ATTR-CA, and imaging techniques such as bone scintigraphy and nuclear imaging are indispensable for diagnosis (Treglia et al., 2018[132]). Galectin-3 (Gal-3) levels were found to be elevated in all amyloidosis groups. However, Gal-3 was unable to differentiate between individuals with cardiac involvement and those without it (Swiger et al., 2016[129]).

A systematic review also highlighted the role biomarkers play in diagnosing and staging CA, with investigators focusing on quantification and standardization of amyloid burden. The review points to the potential of combining imaging techniques and biomarkers for the early detection and monitoring of CA (Kyriakou et al., 2018[70]). Further, a retrospective study found that elevated cardiac troponin I (cTNI) levels were independently associated with reduced survival in cardiac amyloidosis. The study followed 62 patients diagnosed with CA from January 1999 to December 2011, finding that at the three-year follow-up, cardiac amyloidosis patients had significantly higher mortality rates than those without cardiac involvement. The study identified old age, elevated cTNI, left ventricular (LV) diastolic dysfunction and systolic dysfunction, and as all independently associated with reduced survival in cardiac amyloidosis (Lee et al., 2013[73]).

In summary, biomarkers such as hs-cTn and NT-proBNP have proven useful in diagnosing and prognosticating cardiac involvement in systemic light chain amyloidosis. Further, biomarkers and imaging techniques play a complementary role in diagnosing and staging CA, with investigators focusing on standardization and quantification of amyloid burden. Monitoring biomarker levels in CA patients can also aid in measuring treatment efficacy and overall disease status. The use of biomarkers and imaging techniques in CA diagnosis is an active area of research, with much promise for early detection and improved outcomes. A summary of these biomarkers is described in Table 1(Tab. 1).

## Artificial Intelligence

It is challenging to detect rare cases of cardiac amyloidosis (CA) as the symptoms and signs are often similar to those of more common ailments, which results in the delayed application of appropriate treatments. In such situations, Artificial Intelligence (AI) has the potential to serve as a valuable tool for early diagnosis (Goto et al., 2021[50]).

ML [machine learning] models are capable of sourcing the appropriate data from the Electronic Health Record (EHR) systems and can skilfully examine the intricate interactions among numerous input factors (e.g., ICD codes), in an entirely automated way. This process would be less time-consuming than traditional statistical strategies and can also furnish clinical decision aid for physicians or be employed as web-based risk estimators (Huda et al., 2021[57]).

In a research study, the discovery of CA was done with the help of Artificial Intelligence models and ECG or echocardiogram as inputs. The ECG model had a good precision rate based on the C-statistics from the ECG-test set from different medical facilities. The examination of cardiac amyloidosis types indicated a better performance of the model on ATTR amyloid in contrast to AL amyloidosis (Goto et al., 2021[50]).

Examining the global longitudinal strain (GLS) and left ventricular ejection fraction (LVEF) is a critical part of diagnosing cardiac amyloidosis (CA). The research was conducted to analyze if an automated artificial intelligence (AI) technique of gauging global longitudinal strain (GLS) and left ventricular ejection fraction (LVEF) could match estimations and detect anomalies in agreement with standard manual procedures in patients with pre-clinical and clinical cardiac amyloidosis (CA). The study's findings indicated that there were no significant distinctions between manually measured and automatically calculated values of left ventricular ejection fraction (LVEF) and global longitudinal strain (GLS) during both pre-CA and CA stages at the time of diagnosis. The application of automated LVEF and GLS could allow for a quicker evaluation in diverse medical conditions with similar exactness and repeatability to manual techniques (Cotella et al., 2023[22]).

Combining AI with sophisticated cardiovascular imaging provides more reliable outcomes without the utilization of contrast agents. Zhang et al. created a Cardiac Magnetic Resonance (CMR) Virtual Native Enhancement (VNE) imaging system powered by artificial intelligence to detect fibrosis in myocardium without using any contrast agents. This new approach was tested on 1348 people with hypertrophic cardiomyopathy, and they had both late gadolinium enhancement (LGE) scans with contrast and VNE scans. The two methods were found to be in good agreement, and the VNE images were of higher quality than the LGE. Furthermore, VNE images can be created in less than one second, reducing the routine CMR protocol for cine and tissue characterization to fifteen minutes (Zhang et al., 2021[145]).

A normal echocardiogram consists of approximately 70 videos taken from various perspectives, and the viewpoints are not marked in every examination. This means that physicians have to go through all the important features in a lot of videos in every study, and this task can be made simpler with the help of artificial intelligence models. Additionally, measurements can fluctuate from one video to another due to the intrinsic variability in cardiac performance and the issue of representing a 3D object with 2D cross-sectional images. Considering the extent of this variability and the vast amount of information present in each study, which often goes unnoticed, it can be said that echocardiography can benefit from automated learning to facilitate the interpretation (Zhang et al., 2018[144]).

Scientists have shown that incorporating machine learning (ML) algorithms into a cardiovascular risk assessment tool, which combines standardized peri-coronary fat attenuation index (FAI) mapping with clinical risk factors and plaque metrics, is a highly accurate method for predicting the prognosis of cardiovascular risk. A diagnosis of all subtypes of cardiac amyloidosis can be made through observation of apple-green birefringence of endomyocardial biopsy sample under polarized light with Congo red staining, mass spectrometry typing or immunohistochemistry. While a positive extracardiac biopsy sample has to be supported by the typical features in cardiac imaging for a diagnosis to be reached (Goto et al., 2021[50]).

## Treatment Modalities

Over the last ten years, significant progress has been made in the diagnosis and treatment of various types of amyloidosis. Notably, the introduction of a gene-editing technique utilizing the CRISPR-Cas9 nuclease has been a ground-breaking development in this field (Sethi et al., 2023[122]). A comprehensive treatment approach is needed for CA patients, including supportive care and specific treatment for the subtype of amyloidosis. Even though all type of amyloidosis leads to the deposition of fibrils in heat, their specific management is quite different.

The mainstay therapy of supportive care is aimed at symptomatic relief. This includes therapies for congestive heart failure symptoms, arrhythmias, and conduction disorders.

**Heart failure**: Initially, it is recommended to implement general measures to manage symptoms of heart failure (HF). These measures include providing diet counselling, restricting sodium (salt) intake, and managing weight. The mainstay treatment for CA patients with heart failure in CA is loop diuretics (furosemide, torsemide) along with aldosterone antagonists (Eplerenoneor spironolactone). Conventional treatment of HF is not used because of the following reasons -

- Angiotensin-converting enzyme inhibitors (ACE inhibitors) and angiotensin receptor blockers are associated with a higher risk of severe hypotension, particularly in AL amyloidosis patients. This is because the impaired sympathetic nervous system function in these individuals leads to a greater reliance on angiotensin receptors for vascular tone regulation (Selvanayagam et al., 2007[121]).
- Digoxin is avoided as it has a high affinity for the amyloid fibrils and can lead to arrhythmias (Selvanayagam et al., 2007[121]).
- CCBs are also not used because of their negative inotropic effect and affinity for fibrils (Cassidy, 1961[16]; Fikrle et al., 2013[38]).

**Atrial fibrillations and arrhythmias**: According to Sanchis et al. (2019[119]) the overall prevalence of AF in a population of CA patients is significantly higher (44 %) compared to (1 %) in the community. They also reported that the patients with the wtATTR variant had a 71 % prevalence of AF compared to 26 % of AL variant patients. In a study conducted by Longhi et al. (2015[78]) it was found that out of 262 patients with cardiac amyloidosis, 15 % had atrial fibrillation (AF) overall. Among those with wild-type transthyretin amyloidosis (wtATTR), 40 % had AF, whereas only 9 % of patients with AL amyloidosis had AF.

Cardiac amyloid patients often have poor tolerance to most rate control agents such as beta-blockers, calcium channel blockers, and digoxin. However, if rate control is attempted, experts advise starting with low doses and closely monitoring hemodynamics. In the case of digoxin, regular monitoring of drug levels, electrolytes, and renal function is recommended (Muchtar et al., 2018[92]). If atrial fibrillation (AF) becomes refractory, atrioventricular nodal ablation and permanent pacemaker implantation may be considered. It is important to note that cardiac amyloidosis carries an elevated risk of intracardiac thrombus, which can be as high as 33 % (El-Am et al., 2019[35]). Consequently, consensus and guidelines recommend anticoagulation when AF is present, regardless of the CHA2DS2-VASc risk score. Direct oral anticoagulants have been approved as the first-line anticoagulation therapy for cardiac amyloidosis (Kittleson et al., 2023[66]). If patients remain symptomatic despite attempts at rate control, rhythm control strategies may be considered. Amiodarone is the initial choice for rate control (Giancaterino et al., 2020[44]).

**Conduction disorders**: Conduction disorders in cardiac amyloidosis mostly involve the His-Purkinje system (Reisinger et al., 1997[109]). The most commonly indicated management for conduction disorder in CA is permanent pacemaker implantation and cardiac resynchronization therapy. 

In a study conducted by Donnellan et al. it was observed that the implementation of cardiac resynchronization therapy led to enhancements in several aspects, including the severity of mitral regurgitation, NYHA functional class, and left ventricular ejection fraction. (Donnellan et al., 2019[27]).

## Specific Therapy for AL Amyloidosis

Chemotherapy followed by stem cell transplantation is the preferred treatment option for individuals diagnosed with AL amyloidosis, and in some cases, proteasome inhibitors are used. The current standard of therapy is based on antimyeloma therapies and focuses on achieving a significant hematological response. The effectiveness of a treatment is evaluated based on the hematologic and organ response.

The criteria for this response is described in Table 2(Tab. 2).

### Stem cell transplantation

SCT (stem cell transplantation) has shown promising results in terms of hematological and cardiac response rates, with rates of 66 % and 41 % respectively. The most frequently employed treatment approach is administering a high dose of melphalan, followed by autologous stem cell transplantation (HDM/ SCT). Furthermore, there has been a decrease in mortality associated with the transplantation procedure. 

**Alkylating agents**: Alkylating agents used are melphalan, cyclophosphamide, and bendamustine. The most commonly used is melphalan. A high dose of melphalan (100 to 200 mg/m^2^ of intravenous dose divided over 2 days) is given prior to SCT (Comenzo et al., 1996[20]; Moreau et al., 1998[91]).

In cases where SCT is not viable, oral melphalan can be combined with dexamethasone for treatment. Cyclophosphamide, however, tends to be administered alongside proteasome inhibitors and CD38 monoclonal antibodies despite potential adverse effects such as hemorrhagic cystitis and hair loss. Bendamustine serves mainly as a tertiary line of rescue therapy coupled with dexamethasone (Lentzsch et al., 2020[75]).

**Immunomodulators**: Immunomodulatory medications such as thalidomide, lenalidomide, and pomaidomide are currently utilized to treat AL amyloidosis. These drugs work by inhibiting the activity of interleukin-6, which is a growth factor that promotes the proliferation of myeloma cells (Anderson 2005[3]). Lenalidomide in conjunction with dexamethasone is commonly prescribed; however, Pomalidomide - a newer analog of ThalIdoMide - has been found to possess more potent antimyeloma properties than lenalidomide. Ongoing trials aim to determine their optimal utilization in treating AL amyloidosis patients.

**Proteosome inhibitors**: Cell cycle arrest and cellular apoptosis can be induced by inhibiting proteasome activity, making proliferating malignant cells more vulnerable compared to normal cells (Rajkumar et al., 2005[107]) Bortezomib, classified as a first-generation proteasome inhibitor, is used in therapeutic approaches such as cyclophosphamide-bortezomib-dexamethasone or bortezomib-melphalan-dexamethasone. The second-generation boronic acid dipeptide proteasome inhibitor Ixaxomib exhibits significantly enhanced tumor protease inhibition abilities. Conversely, Carfilzomib represents a relatively more discerning alternative to bortezomib and has demonstrated efficacy against patients who are resistant to bortezomib treatment (Driscoll and Girnius, 2016[31]).

**Monoclonal antibodies**: CD38 and SLAMF7 are cell surface antigens being evaluated as targets for AL amyloidosis as they are specific to MM cells. Daratumumab is an anti-CD38 monoclonal antibody used in the Dara-CyBorD regimen. It has now become the standard method of care for individuals who are newly diagnosed with AL amyloidosis (Staron et al., 2021[127]). Another CD38 antibody that is being tested is Isatuximab. The SLAMF7 antibody that is being tested is Elotuxumab. CD38 is a glycoprotein that spans the cell membrane and interacts with lipid rafts to control calcium movement within cells. Its expression is consistent and prevalent in plasma cells found in individuals with multiple myeloma or amyloid light-chain disease (Lisenko et al., 2016[77]).

**Doxycycline**: Doxycycline is a type of tetracycline antibiotic that inhibits matrix metalloproteinases activity. Doxycycline is categorized as a tetracycline antibody, which hinders the action of Matrix metalloproteinases. Evidence suggests that increased serum levels and tissue expression of MMP and tissue inhibitor of matrix metalloproteinase are linked to renal and cardiac impairment in AL. It suppresses the production of amyloid fibrils formed due to light chains. The effectiveness varies depending on the dosage administered *ex vivo *or* in vivo*. Research studies are currently underway to evaluate its efficacy when used concurrently with SCT.

Other new modalities that are coming up are the

- Small molecule inhibitors - venetoclax. They lead to the modulation of intrinsic apoptotic pathways.
- Clearance of amyloid deposits- 11-1F4 and NEOD001 are being tested on mouse models. These are exogenous antibodies that are targeted to kappa and light chain extracts.

## Specific Therapies for ATTR Amyloidosis

Different type of therapies using different mechanisms of action are present for ATTR type of amyloidosis.

**Stabilizers**: They stabilize the TTR tetramer. These include Tafamidis, Diflunisal, and AG-10. Tafamidis and AG10 are selective stabilizers whereas diflunisal is a non-selective agent. Tafamidis is an FDA-approved drug for ATTR cardiomyopathy. Tafamidis is selective for thyroxine-binding sites of the TTR protein. The Transthyretin Amyloidosis Cardiomyopathy Clinical Trail (ATTR-ACT) evaluated the efficacy and safety in hereditary and wild-type cases. The rate of cardiovascular-related hospitalizations was lower in the treatment, with 32 % less risk when compared to the placebo (Maurer et al., 2018[88]) . Diflunisal is a nonsteroidal anti-inflammatory agent which binds to the T4 binding sites on TTR, though with lower affinity than tafamidis. It is given at a dose of 250 mg orally twice daily, which is below the dose recommended for anti-inflammatory activity and is well tolerated (Castaño et al., 2012[17]) . In a phase 3 trial of 441 patients with wild-type and hereditary ATTR-CA, tafamidis reduced all-cause mortality and cardiovascular-related hospitalizations (Maurer et al., 2018[88]). AG10 is a potent and selective stabilizer of TTR, exceeding the efficacy of tafamidis in stabilizing WT and variant TTR in serum (Penchala et al., 2013[104]).

**Silencers**: These drugs affect the production of TTR by interrupting the stages of protein synthesis on a molecular level. In these, the first-generation drugs are - Inotersen and Patisiran. Patisiran is a small intervering RNA blocking the expression of both variant and wt-TTR. It targets the 3′ untranslated region of the TTR mRNA. In phase 3 trials, patisiran significantly improved neuropathy, leading to a statistically significant reduction in NT-proBNP left ventricular wall thickness and reduced worsening in GLS (suggestive of cardiac benefit) (Adams et al., 2018[1]; Solomon et al., 2019[125]). Inotersen is an antisense oligonucleotide inhibiting the production variant and wtTTR. The NEURO-TTR trial showed that serum TTY reached steady-state levels by 13 weeks, with a mean reduction in serum TTY of 74 % and a median 79 % (Benson et al., 2018[6]). The second-generation drugs are the Vutrisirian. Vutrisirian is siRNA conjugated to GaINAc, which binds to TTR mRNA in the nucleus and initiates mRNA degradation via RNase H2. In the HELIOS-A phase 3 study, vutrisan showed improvement compared with placebo in the exploratory cardiac endpoint of change from baseline in NT- proBNP (Adams et al., 2023[2]).

**Degraders**: These drugs destabilize and disaggregate the amyloid fibers. The combination of doxycycline and tauroursodeozycholic acid (TUDCA) is the most effective regime. Trials have shown that TUDCA combined with doxycycline has shown some ability to decrease left ventricular strain after a year but does not entirely halt the disease (Di Giovanni et al., 2019[25]).

**Liver and cardiac transplantation**: The best treatment for ATTR type of amyloidosis is combined heart and liver transplantation. Fibrils are formed in the liver therefore transplanting the liver would remove the source of mutated TTR molecules and prolongs survival. But this type of transplantation is most feasible in younger patients who got the diagnosis earlier in the disease course.

## Advances in Treatment Modalities

### AL amyloidosis

The latest modalities targeted by monoclonal antibodies are plasma cells. A human monoclonal antibody called daratumumab is directed against CD38, prominently expressed in abnormal plasma cells. Daratumumab demonstrated promising outcomes in early trials in patients with AL amyloidosis. The hematological response was obtained in 76 % of cases (36 % with complete remission) in a retrospectively conducted trial involving 25 patients who had extensive pretreatment with the dosing such as once every week for two months, administering 16 mg/kg weight in 1 l for the initial dose and 500 mL for subsequent doses (Spoladore et al., 2021[126]). Amyloid deposits are also the novel target of monoclonal antibodies. A monoclonal IgG1 kappa antibody which is humanized and called NEOD001 (monthly infusions) has a strong affinity for misfolded light chains. This chemical can lower the amount of amyloid in the body by dissolving soluble light chain aggregates and removing insoluble fibrillar aggregates via ingestion by monocytes and macrophages (Spoladore et al., 2021[126]).

### ATTR amyloidosis

For ATTR amyloidosis, Tafamidis is a tiny medication that binds to the transthyretin's thyroxin binding site, stabilizing TTR tetramers in both wild-type and genetically modified TTR (e.g., V30M, V122I), taken 80 mg (four 20-mg tafamidis meglumine capsules) orally once daily. Tafamidis primarily underwent evaluation in polyneuropathy trials, with encouraging outcomes regarding the degradation of nerve function (Spoladore et al., 2021[126]).

## TTR Silencers

Among the therapeutic agents that target transthyretin mRNA, Patisiran (administered intravenously at a dose of 0.3 mg/kg every three weeks) and Inotersen (subcutaneously administered once a week at a dose of 284 mg) are two oligonucleotide-based drugs currently available. By targeting the 3' untranslated region of transthyretin mRNA, Patisiran acts as double-stranded RNA in circulation but eventually dissociates into single-stranded molecules within cells. The sense strand subsequently binds to TTR mRNA forming an RNA-induced silencing complex resulting in hindering protein synthesis through cleavage inhibition, thereby controlling both normal and mutant TTR protein levels. Similarly, Inotersen is an antisense oligonucleotide that primarily works by binding with its target or complementary sequence on mRNA inside the nucleus followed by degradation facilitated by ribonuclease H enzyme action after translation regulation has been affected effectively ensuring efficient reduction on overall protein production from either normal or pathological variants (Buxbaum, 2018[12]).

## TTR Stabilizers

Tafamidis functions as a particular TTR stabilizer by occupying one of the thyroxine-binding sites present in the TTR tetramer, thereby preventing the initial stage of amyloidogenic cascade dissociation. It has been approved for individuals with polyneuropathy at an oral dosage quantity of 20 mg per day. For those having cardiomyopathy, they are advised to consume a daily dose quantity ranging from 80 mg/61 mg once (Park et al., 2020[101]).

Diflunisal is a type of analgesic medication derived from salicylic acid and is typically taken at 250 mg twice daily. Its mechanism of action involves interfering with the binding site located at the dimer-dimer interface of TTR tetramers, ultimately leading to TTR stabilization. This stabilizing effect induces complete kinetic stabilization in wild-type and variant TTR forms, thus effectively inhibiting fibril formation (Berk et al., 2013[8]). 

Vutrisiran, also known as ALN-TTRsc02, is a newer type of RNAi-based medicine that aims to block the production of transthyretin protein by targeting a specific segment in TTR messenger RNA. This approach works by impeding the formation of TTR protein altogether instead of reducing its levels like many first-generation therapie (Habtemariam et al., 2021[53]).

## CRISPR/Cas9 Gene Editing

The method of gene editing known as Clustered Regularly Interspaced Short Palindromic Repeats/CRISPR-associated protein 9 is capable of cutting DNA and inducing natural DNA repair processes, which can lead to the elimination, deletion or insertion of a specific genetic sequence. In cases related to hereditary amyloidosis caused by transthyretin, this therapy can be used to knock out TTR gene after just one application. NTLA-2001 is an innovative approach based on CRISPR-Cas9 that entails intravenous administration. This treatment targets hepatocyte cells for editing TTR genes resulting in reduction in both mutant and wild-type proteins post single-dose consumption via lipid nanoparticle delivery with liver tissue affinity comprising two components: a unique RNA guide targeting TTR and mRNA sequence originating from *Streptococcus pyogenes* Cas9 protein technology (Gillmore et al., 2021[46]; Sabnis et al., 2018[117]).

A clinical experiment on mice yielded a knockdown rate of more than 97 % of the mouse TTR. LNP-INT01 was given to mice in doses 0.3-3 mg/kg. TTR levels were shown to be consistently lower after a year of follow-up. By day 28, the TTR level decreased by 52 % in the group administered with a dosage of 0.1 mg/kg, while experiencing an 87 % decrease in TTR levels was observed in the group receiving the minimum dose (Finn et al., 2018[39]).

Acoramidis, formerly AG10, is a TTR stabilizer with high selectivity that emulates the configuration of Thr119Met mutation's protective qualities. Its mechanism inhibits tetramer dissociation by encouraging hydrogen bonds to form between serine residues (Judge et al., 2019[59]). This is explained in Table 3(Tab. 3).

Tolcapone can stabilize TTR tetramers by engaging with both binding sites for thyroxine within the TTR tetramer (Gamez et al., 2019[41]).

## TTR Disrupters

Selective targeting of dissociative monomers, oligomers or TTR aggregates by monoclonal antibodies can effectively hinder fibril formation and further aggregation. Additionally, these antibodies can target amyloid deposits which initiate phagocytic mechanisms that spare normal tetrameric TTR from removal (Hosoi et al., 2016[55]).

### Dezamizumab

Dezamizumab, an IgG1 antibody, effectively eliminates amyloid deposits containing SAP by triggering complement activation and macrophage-mediated phagocytosis (Bodin et al., 2010[10]). Research studies have shown that administration of a dose exceeding 1200 mg can significantly reduce kidney-related amyloid build-up; over 2000 mg has been observed to be effective in degrading liver-based build-ups caused due to the accumulation of these harmful protein aggregates (Richards et al., 2018[112]).

### Doxycycline and tauroursodeoxycholic acid

It has been observed that they can interfere with TTR fibrils in amyloid deposits present in ATTRv and -wt patients. Although doxycycline, a derivative of tetracycline, is demonstrated to be the most effective method for disrupting pre-existent TTR fibrils, it was not completely successful with non/pre-fibrillar TTR deposits. Strong synergistic effects were noted when combining both doxycycline and tauroursodeoxycholic acid, which facilitated destruction and absorption of the deposited TTR molecules (Cardoso et al., 2003[15]). A phase 2 investigation administered daily oral doses of 100 mg of doxycycline together with triple dosages (3*250 mg) of tauroursodeoxycholic acid to ATTRv and -wt patients; as a result, stable disease was reported over a course lasting approximate duration up until one year (Obici et al., 2012[98]).

## Conclusion

Due to the diverse pathogenesis of CA, it often presents with various clinical features, making it hard to diagnose. This is further complicated by its similarity to more common disorders. However, owing to the advances in diagnostic techniques, drug therapies, and AI, it is now possible to diagnose early and begin the treatment immediately. In light of the current awareness of the significance of ATTR-CM, medical professionals should be on the lookout for cardiac amyloidosis when patients show symptoms of CA with adequate clinical suspicion. Additionally, serologic markers testing, nuclear imaging, and tissue biopsy should be employed promptly to confirm the diagnosis and identify the type of CA. Endomyocardial biopsy is particularly important when the subtype of CA is unclear or suspicion persists despite the results of serologic and imaging tests. All diagnostic methods should be used together to ensure the most accurate diagnosis. Risk assessment for unfavorable outcomes can be achieved by utilizing various imaging modalities. Furthermore, certain CA biomarkers, such as troponin and NT-proBNP, also help in risk stratification and monitoring the effectiveness of treatment outcomes in CA patients. Diagnosing CA and differentiating its subtype is necessary to begin a suitable therapeutic regimen with disease-modifying agents.

Several problems regarding the clinical approach to CA-including uncertainties in screening, progression evaluation, AT-TRv asymptomatic carriers management, and the changes that should be done with treatment strategies for non-responders-have yet to be resolved. Additionally, the most effective treatment, single therapy or a combination of gene silencer and stabilizer is currently purely based on the clinical judgement of the physician. More research is required to identify the optimal medical treatment for ATTR-CM ensuring affordability and accessibility.

## Notes

Mohammad Amjad Kamal and Nirja Kaka (Pear Research, Dehradun, India; E-mail: nirja@pearresearch.com) contributed equally as corresponding author.

## Declaration

### Acknowledgments

The study was conducted under the supervision of PearResearch (pearresearch.com). 

### Conflict of interest 

The authors declare no conflict of interest. There are no relevant financial or non-financial competing interests to report. 

### Ethical considerations

Not applicable.

### Funding

The study did not receive any funding from any authority or organization.

### Availability of data and material

Not applicable.

### Authors' contributions

NK, NP, YS: Conceptualization, methodology, project administration, resourses, and supervision. All authors: Visualization, roles/writing; MAK, NK, NP, YS: Writing - review & editing.

## Figures and Tables

**Table 1:**
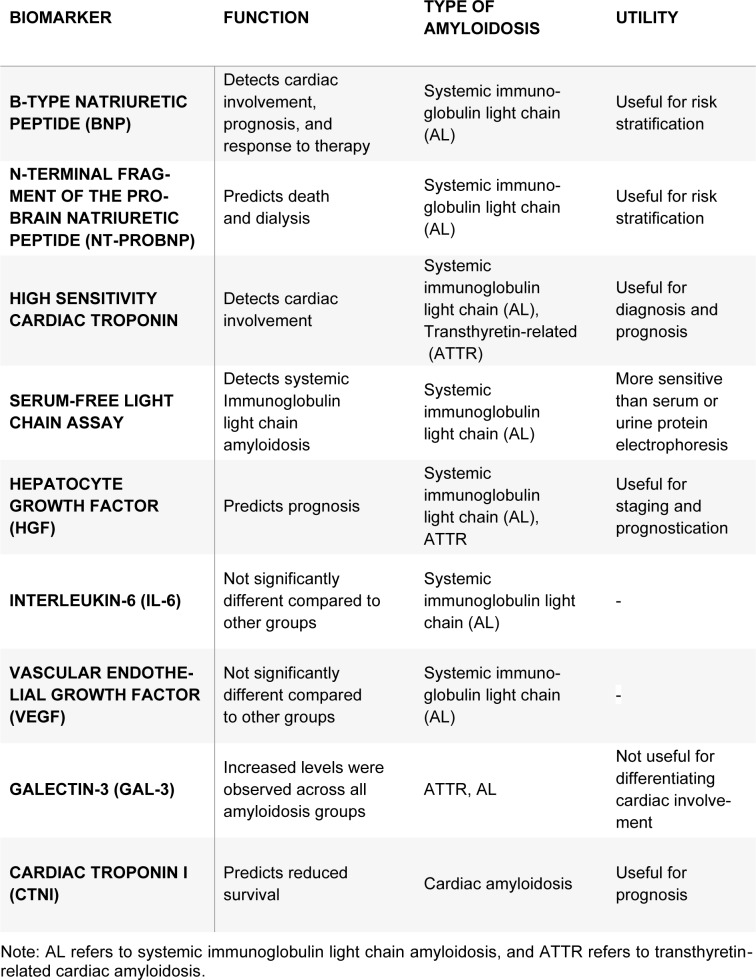
Biomarkers for diagnosing cardiac amyloidosis

**Table 2:**
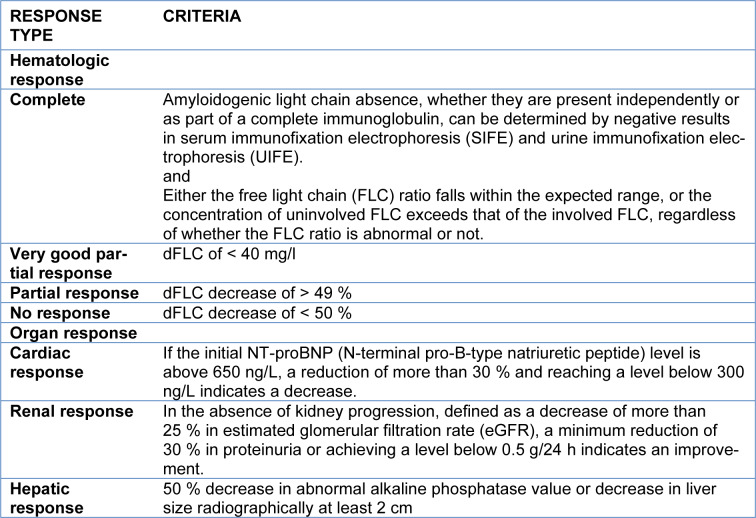
Criteria for response to therapy

**Table 3:**
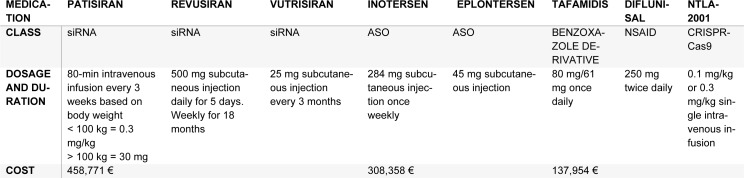
Medication with their usage

**Figure 1:**
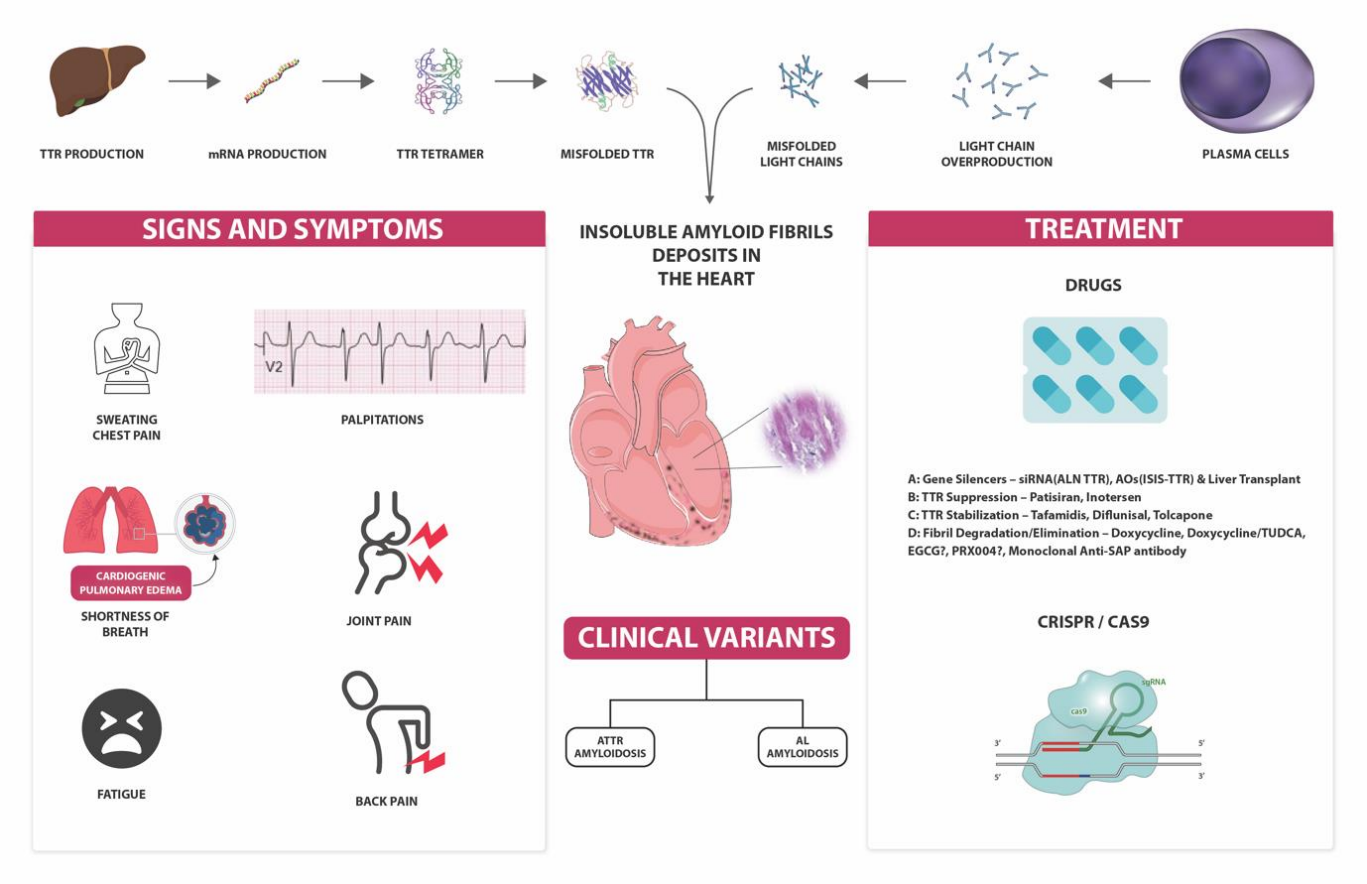
Graphical abstract “Cardiac amyloidosis” (made by pearresearch (pearresearch.com))
